# Supplementary material for: NanoLoop: A Deep Learning Framework Leveraging Nanopore Sequencing for Chromatin Loop Prediction
Source: Adv Sci (Weinh). 2026 Feb 13;13(24):e17692. doi: 10.1002/advs.202517692 (PMC13116353; doi:10.1002/advs.202517692)
Supplement: Supplementary file 1 — Supporting File 1: advs74349‐sup‐0001‐SuppMat.docx. [file ADVS-13-e17692-s001.docx]

**Supplementary Figures**


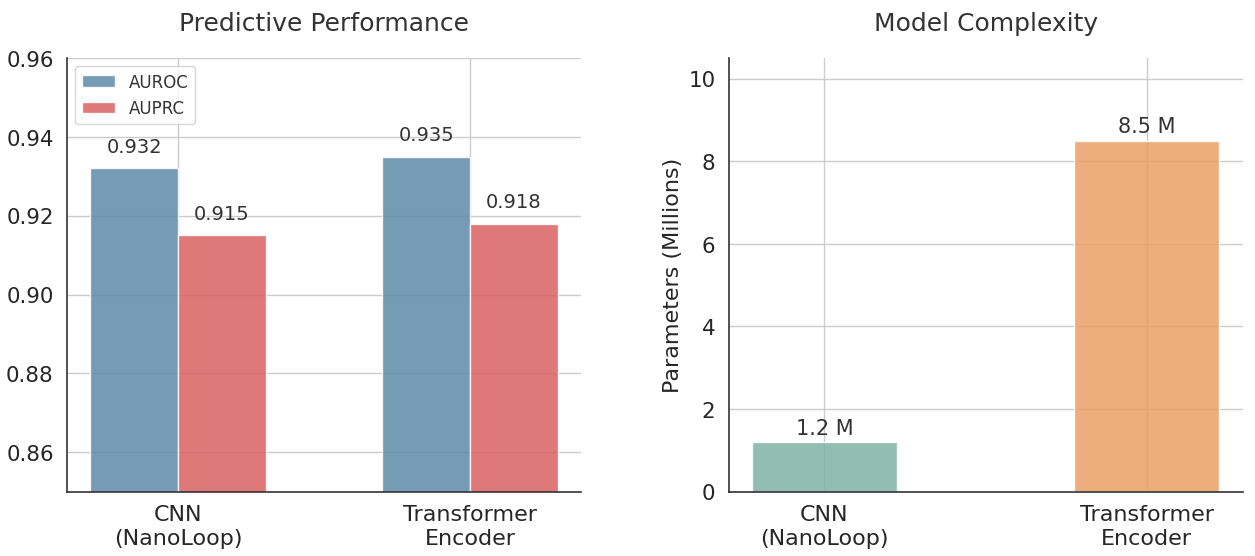


**Supplementary Fig. S1. Comparison of predictive performance and computational efficiency between CNN and Transformer architectures.** (**A**) Precision-Recall curves showing the model results when using a CNN-based sequence module versus a Transformer-based module. (**B**) Comparison of the number of model parameters between the CNN and Transformer architectures.


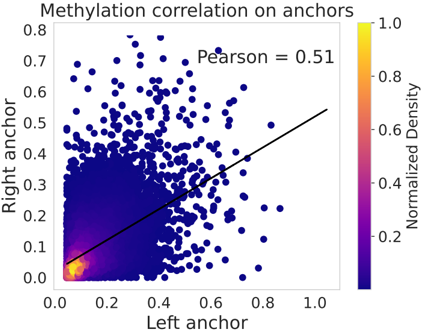


**Supplementary Fig. S2. Distribution of DNA methylation levels across anchor pairs.** Each point represents a loop, with the x- and y-axes indicating the methylation levels of the left and right anchors, respectively. The overall Pearson correlation coefficient for anchor pair methylation levels is 0.51.


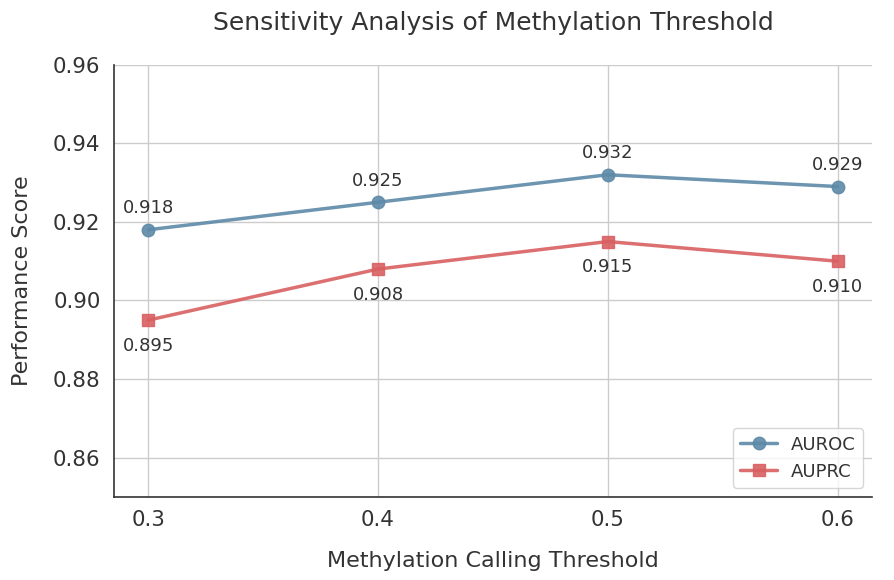


**Supplementary Fig. S3. Sensitivity analysis of NanoLoop performance across different methylation calling thresholds.** Model performance across varying methylation probability thresholds (0.3–0.6), with the optimal predictive accuracy achieved at a threshold of 0.5.


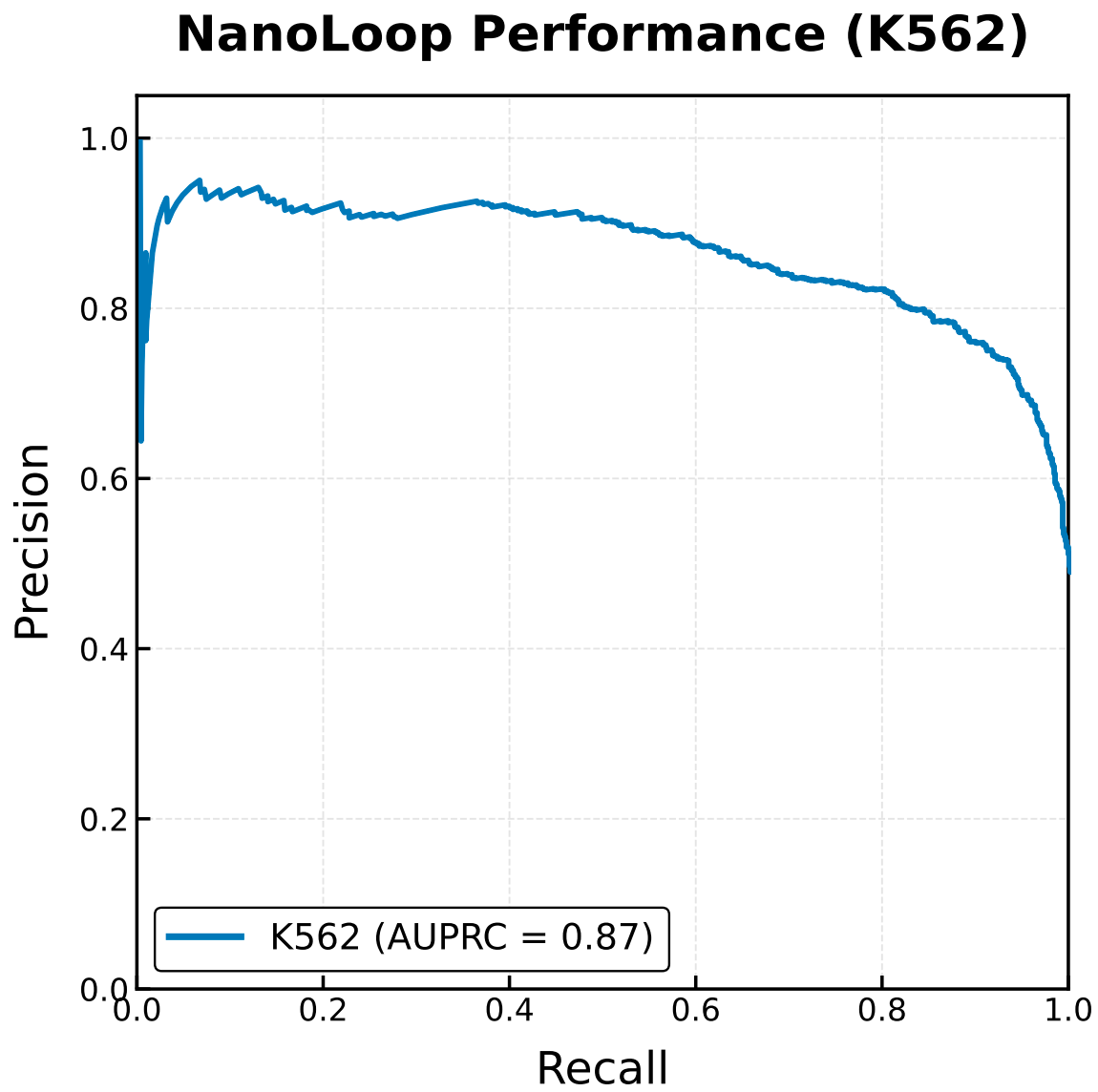


**Supplementary Fig. S4. Generalizability of NanoLoop performance on the K562 cell line.**


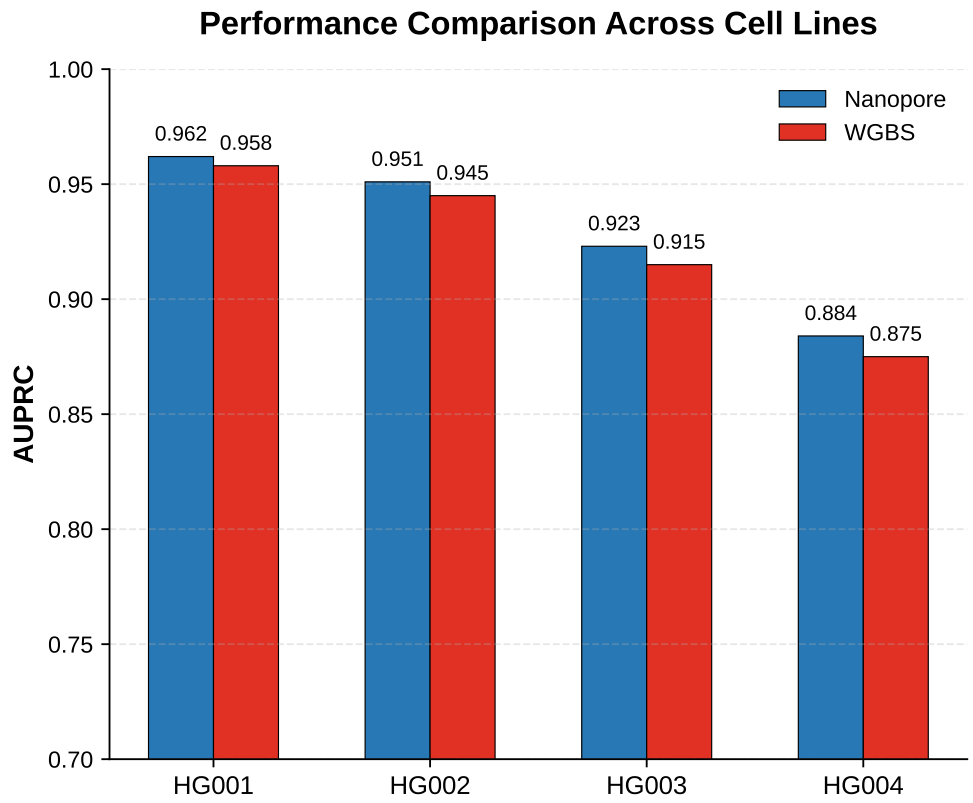


**Supplementary Fig. S5. Performance comparison of NanoLoop using Nanopore-derived vs. WGBS-derived methylation data.**The bar chart illustrates the AUPRC performance of NanoLoop when utilizing native Nanopore methylation signals versus traditional WGBS methylation signals across four distinct lymphoblastoid cell lines (HG001, HG002, HG003, and HG004).


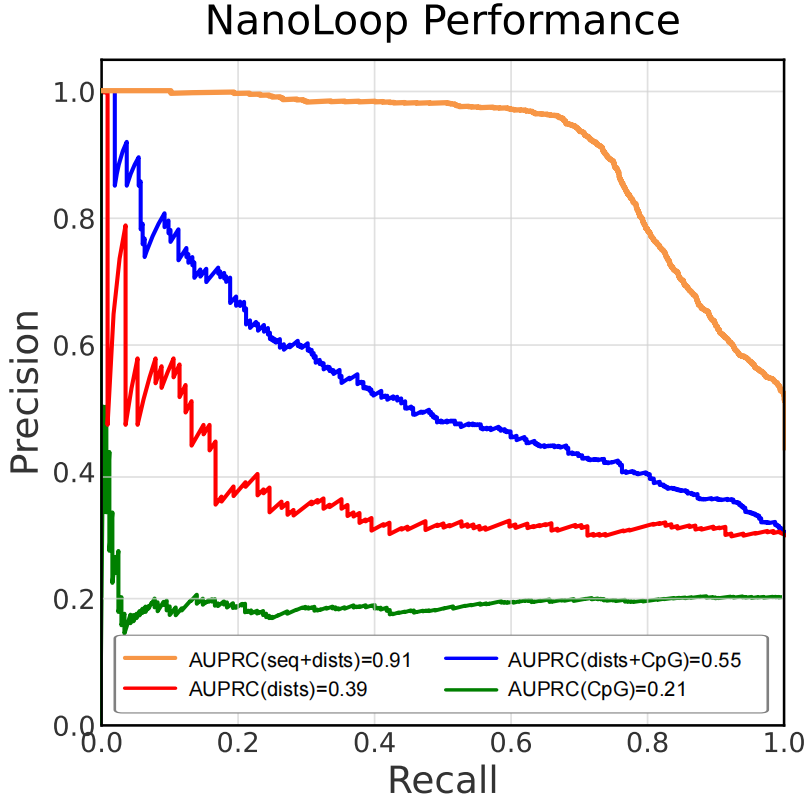


**Supplementary Fig. S6. Systematic ablation study and baseline comparison of NanoLoop feature combinations.**The Precision-Recall curves illustrate the predictive performance of various feature sets to evaluate their relative contributions: (1) CpG (methylation) only, (2) dists (genomic distance) only, (3) dists + CpG, and (4) seq + dists (sequence and distance).


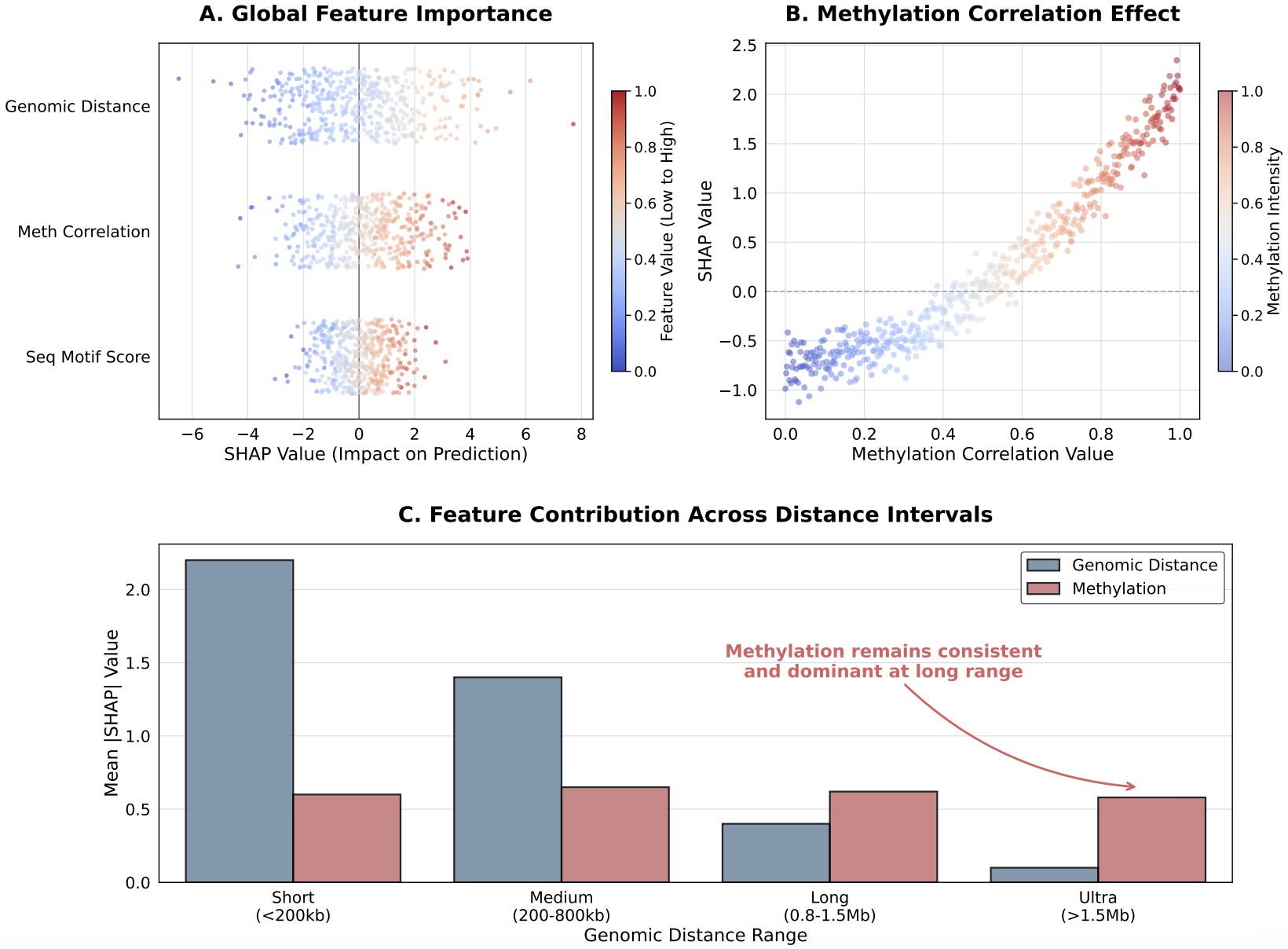


**Supplementary Fig. S7. Mechanistic interpretation of NanoLoop decisions via SHAP analysis. (A)** Global feature importance. Summary of feature rankings based on mean absolute SHAP values, illustrating the relative contribution of genomic distance, methylation correlation, and sequence features to model predictions. (**B**) SHAP dependence for methylation correlation. A scatter plot showing the relationship between methylation correlation values at loop anchors and their corresponding SHAP values. (**C**) Distance-stratified feature contribution. Analysis of SHAP values for genomic distance (blue) and methylation correlation (red) across different genomic distance intervals ranging from <200 kb to >1.5 Mb.


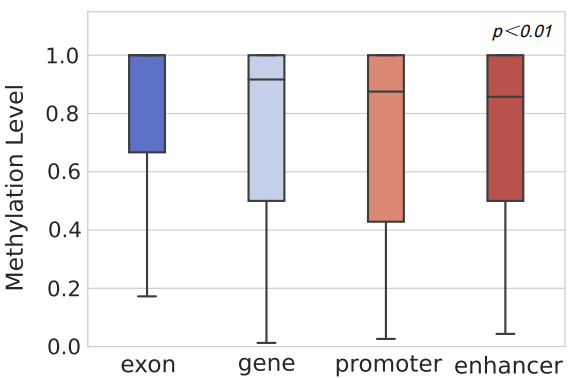


### **Supplementary Fig. S8.** **Methylation status of regulatory elements.** The methylation probability of CpG sites within exon, gene, promoter, and enhancer regions was calculated as the methylation level (p < 0.01, Wilcoxon rank-sum test).


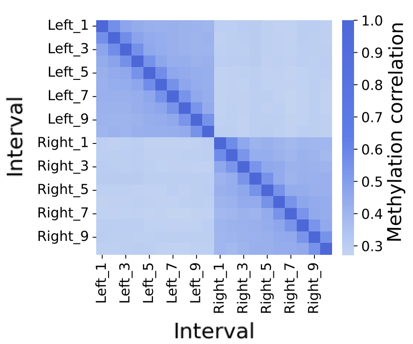


### **Supplementary Fig. S9. Correlation of average methylation levels across different bins of anchor pairs.** Left_1 to Left_10 represent the left anchor evenly divided into 10 intervals, and Right_1 to Right_10 represent the right anchor similarly. The methylation level is calculated as the proportion of methylation sites with a frequency ≥0.5 in each interval, and the methylation correlation between intervals is then computed.


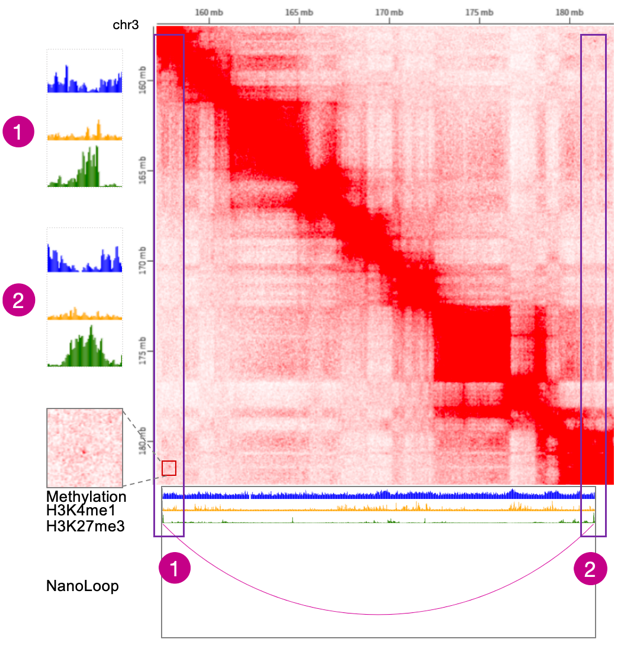


### **Supplementary Fig. S10. NanoLoop-Predicted Long-Range Chromatin Loops on Chromosome 3 with Epigenetic Signal Enrichment.** Hi-C heatmap showing a NanoLoop-predicted long-range chromatin loop on chromosome 3 in HSPCs, spanning about 25 Mb. Tracks 1 and 2 display reduced methylation and H3K27me3 enrichment at loop anchors.


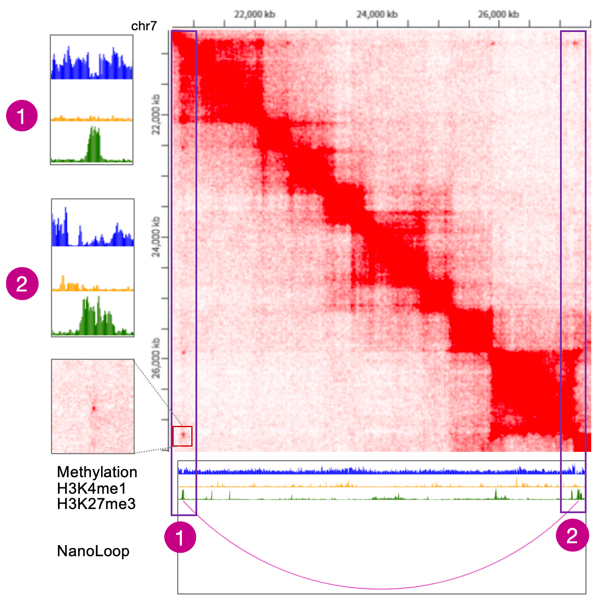


### **Supplementary Fig. S11. NanoLoop-Predicted Long-Range Chromatin Loops on Chromosome 7 with Epigenetic Signal Enrichment.** Hi-C heatmap showing a NanoLoop-predicted long-range chromatin loop on chromosome 7 in HSPCs, spanning about 6 Mb. Tracks 1 and 2 display reduced methylation and H3K27me3 enrichment at loop anchors.


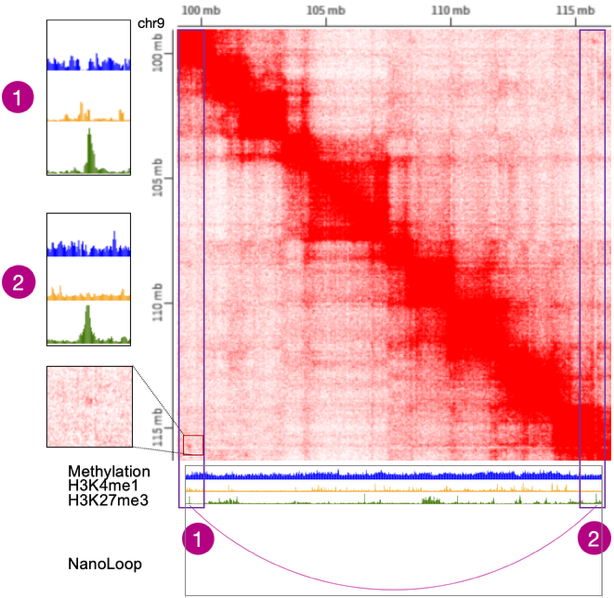


### **Supplementary Fig. S12. NanoLoop-Predicted Long-Range Chromatin Loops on Chromosome 9 with Epigenetic Signal Enrichment.** Hi-C heatmap showing a NanoLoop-predicted long-range chromatin loop on chromosome 9 in HSPCs, spanning about 15 Mb. Tracks 1 and 2 display reduced methylation and H3K27me3 enrichment at loop anchors.
